# Supplementary material for: Tetraspanin 4 stabilizes membrane swellings and facilitates their maturation into migrasomes
Source: Nat Commun. 2023 Feb 23;14:1037. doi: 10.1038/s41467-023-36596-9 (PMC9950420; doi:10.1038/s41467-023-36596-9)
Supplement: Supplementary file 1 — Supplementary Information [file 41467_2023_36596_MOESM1_ESM.pdf]

Supplementary information

Tetraspanin 4 stabilizes membrane swellings and facilitates their maturation into migrasomes

Raviv Dharan*[1,2], Yuwei Huang*[3], Sudheer Kumar Cheppali[1,2], Shahar Goren[1,2,4], Petr Shendrik[1], Weisi Wang[3], Jiamei Qiao[3], Michael M. Kozlov[2,5], Li Yu[6], Raya Sorkin[1,2]

1. School of Chemistry, Raymond & Beverly Sackler Faculty of Exact Sciences, Tel Aviv University, Israel
2. Center for Physics and Chemistry of Living Systems, Tel Aviv University, Tel Aviv, Israel
3. School of Basic Medical Sciences, Xi'an Jiaotong University, Xi'an 710049, China
4. School of Mechanical Engineering, The Ivy and Aladar Fleischman Faculty of Engineering, Tel Aviv University
5. Department of Physiology and Pharmacology, Sackler Faculty of Medicine, Tel Aviv University, Tel Aviv, Israel
6. The State Key Laboratory of Membrane Biology, Tsinghua University-Peking University Joint Centre for Life Sciences, Beijing Frontier Research Center for Biological Structure, School of Life Sciences, Tsinghua University, Beijing 100084, China

* These authors contributed equally

Corresponding author: Raya Sorkin rsorkin@tauex.tau.ac.il

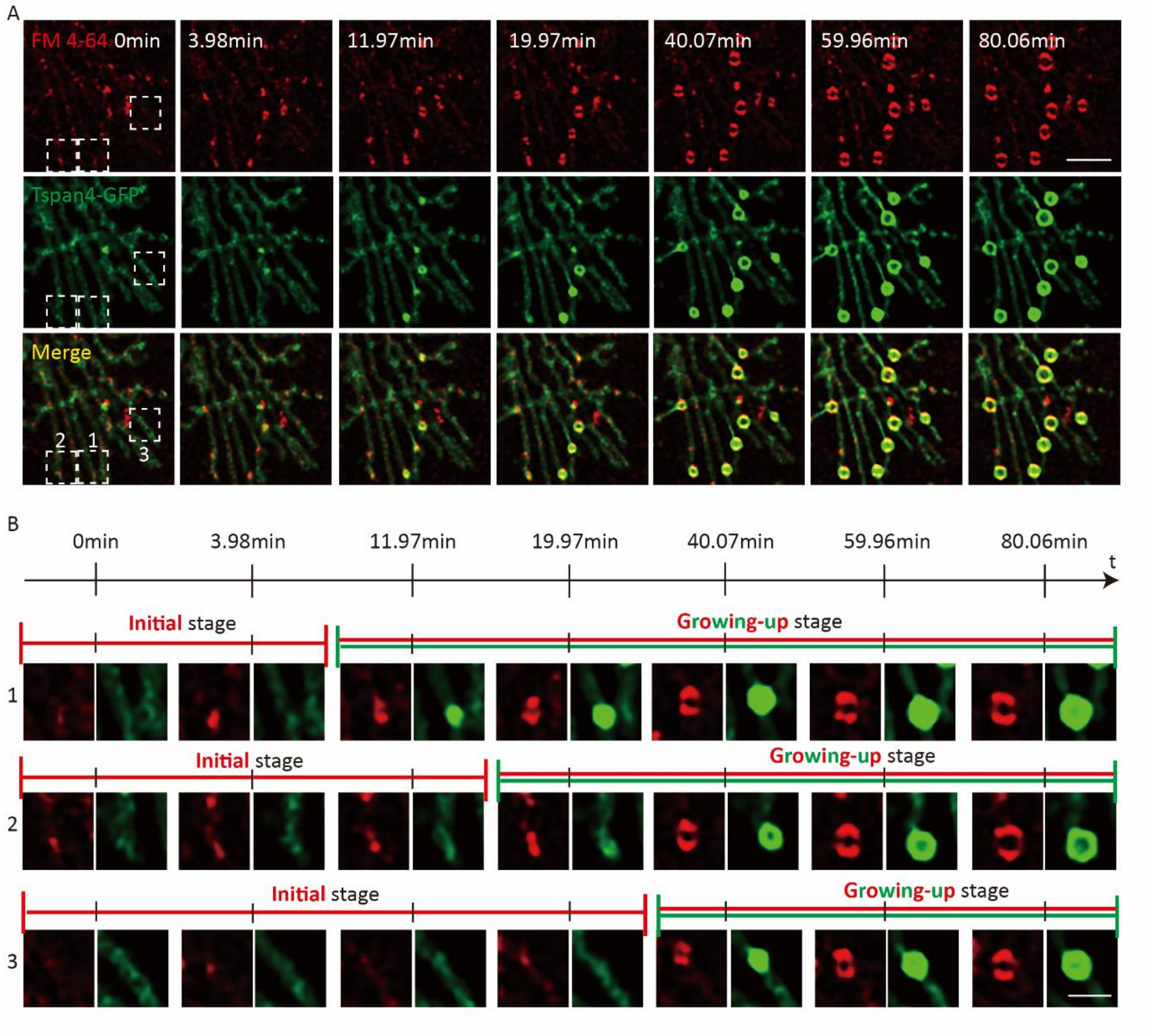

Figure S1. (A) Confocal Time-lapse images of NRK TSPAN4-GFP cells stained by FM4-64 (5 μg/mL). Green, TSPAN4-GFP; red, FM4-64. Scale bar, 5μm. (B) Representative migrasome 1, 2 and 3 selected from A, zoomed in. The initial stage and growing stage are shown. Scale bar, 2 μm.

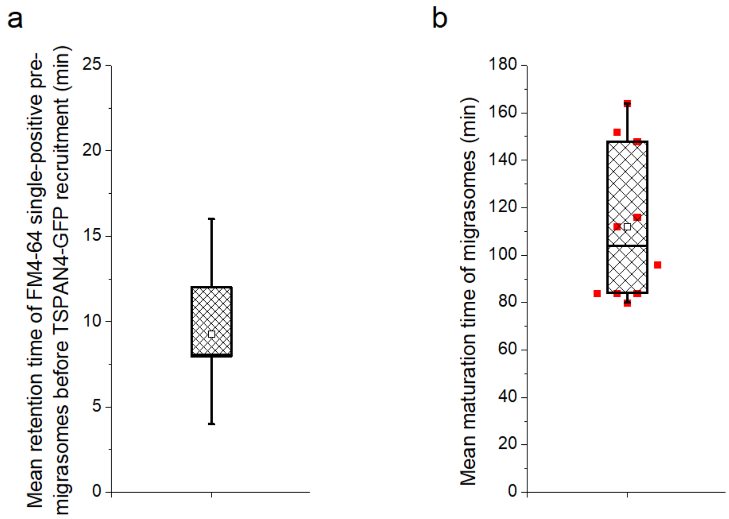

Figure S2. Mean time of mature migrasome formation. (A) Box plot of the mean retention time of FM4-64 single-positive pre-migrasomes before TSPAN4-GFP recruitment. From bottom to top horizontal lines correspond to 5%, 25%, 50%, 75% and 95%, the square represents the mean (9.2 ± 0.5 min). N=66 pre-migrasomes from 10 NRK-TSPAN4 cells from 4 independent experiments. (B) Blox plot with individual data points of the mean maturation time of red-yellow migrasomes (first enriched with FM4-64 and then enriched with FM4-64 and TSPAN4-GFP) defined as in Figure 1D. From bottom to top horizontal lines correspond to 5%, 25%, 50%, 75% and 95%, the square represents the mean (112 ± 10 min). N=10 migrasomes from 10 NRK-TSPAN4 cells from 3 independent experiments. Source data are provided as a Source Data file.

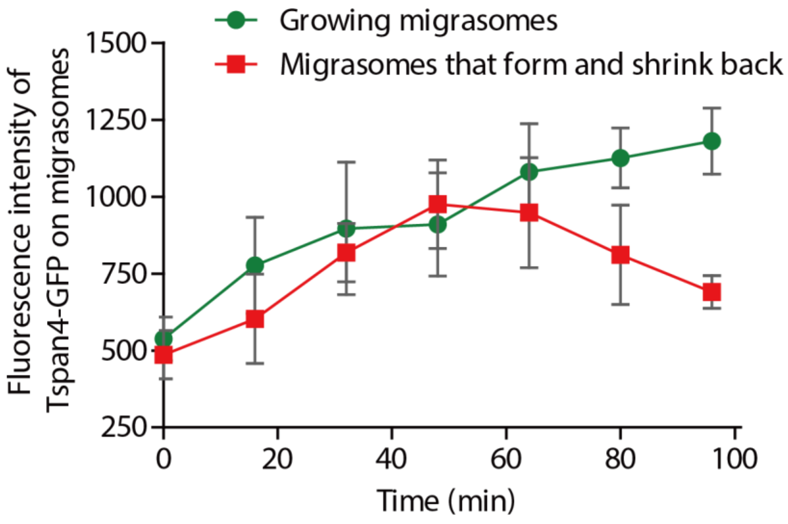

Figure S3. TSPAN4 fluorescence in growing vs shrinking migrasomes shown in Figure 1E. Quantification of TSPAN4-GFP intensity on four growing migrasomes or four migrasomes that form and shrink back shown in Figure 1E indicated by yellow or white arrows, respectively. Error bars are SEM. Source data are provided as a Source Data file.

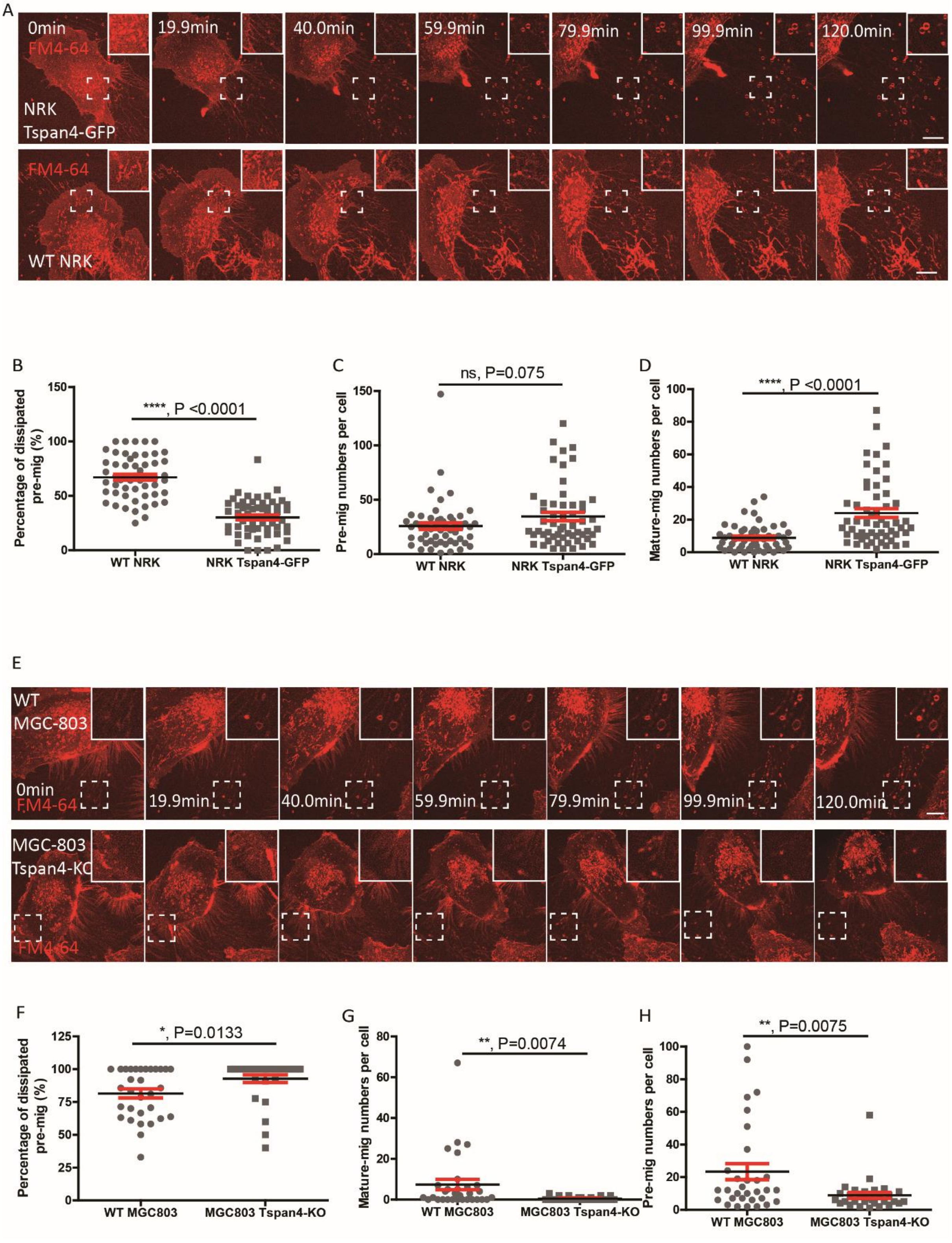

Figure S4. (A) Time-lapse images of NRK Tspan4-GFP cells and WT NRK cells. Cells were pre-stained by FM4-64, then observed by confocal microscope. Scale bar, 5 µm. (B) Statistics of percentage of dissipated pre-migrasomes. P value = 0. (C) Statistics of pre-migrasome numbers per cell. (D) Statistics of mature-migrasome numbers per cell. P value = $1.6x10^{-6}$ (B),(C),(D): N=54 cells, including 1391 pre-migrasomes for WT NRK group and N=55 cells, including 1899 pre-migrasomes for NRK Tspan4-GFP group from 4 independent experiments.

(E) Time-lapse images of WT MGC803 cells and MGC803 Tspan4-KO cells. Cells were pre-stained by FM4-64, then observed by confocal microscope. Scale bar, 5 µm. (F) Statistics of percentage of dissipate pre-migrasomes. (G) Statistics of mature-migrasome numbers per cell. (H) Statistics of pre-migrasome numbers per cell. (F),(G),(H): N=31 cells, including 724 pre-migrasomes for WT MGC803 group and N=31 cells, including 274 pre-migrasomes for MGC803 Tspan4-KO group from 4 independent experiments. All error bars are mean ± SEM. Two-tailed unpaired t-tests were used for statistical analysis. Source data are provided as a Source Data file.

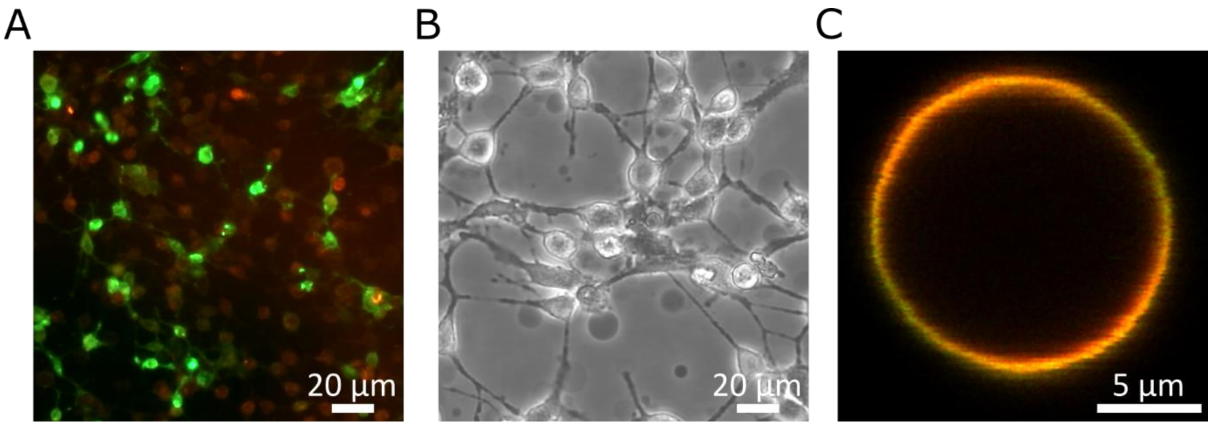

Figure S5. Formation of giant plasma membrane vesicles (GPMVs) from transfected NRK cells expressing TSPAN4-GFP. (A) Microscopy image of NRK cells expressing TSPAN4-GFP (green) and dyed with the membrane dye DIi-C12 (red). (B) Microscopy image of NRK cells expressing TSPAN4-GFP and labeled with DiI-C12, after treatment with a vesiculation buffer. GPMVs, which appear dark in phase contrast image, can be seen floating in the sample or attached to the cells. (C) Confocal microscopy image of GPMVs containing TSPAN4-GFP and DiI-C12. The experimental procedure shown in A-C was repeated independently 4 times (for NRK-TSPAN4-GPMVs) with similar results.

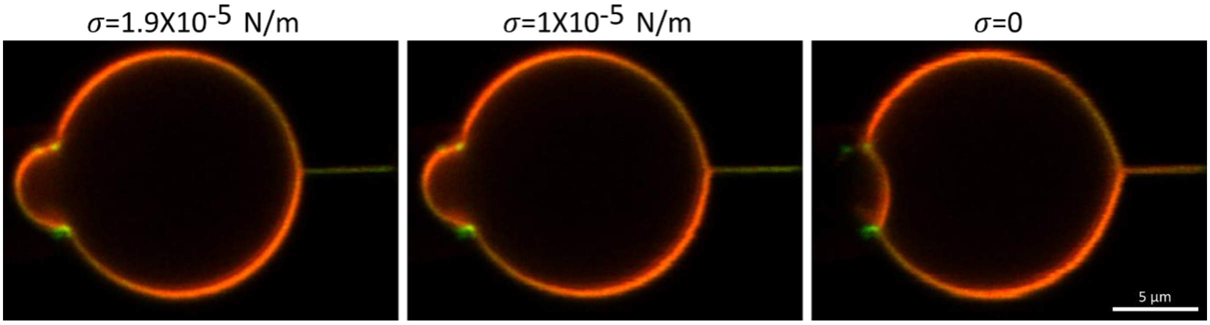

Figure S6. Membrane tube pulling from aspirated NRK-GPMV labeled with DiI-C12 (red) and containing TSPAN4-GFP (green). In this setup the GPMV is a model for the cell and the membrane tube imitates a retraction fiber. By changing the suction pressure in the micropipette, we can control the membrane tension (σ) and hence the tube diameter i.e., the membrane curvature of the tube. Because TSPAN4 is a positive membrane curvature sensor[1], high membrane tension induces TSPAN4 enrichment in the tube. The same results were reproduced for 19 membrane tubes pulled from 9 vesicles (NRK-TSPAN4-GPMVs) in 4 independent experiments.

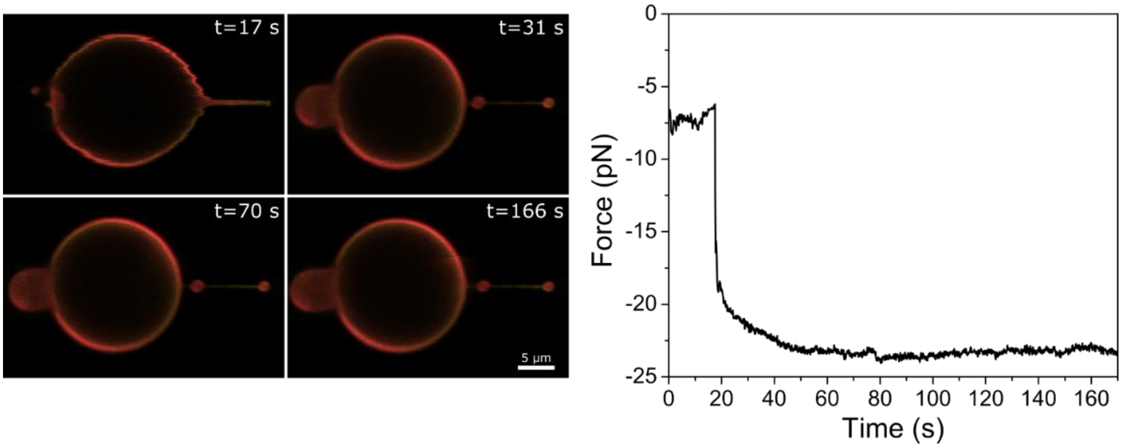

Figure S7. Swelling formation in HEK293T-GPMVs induced by tension increase. On the left time-lapse confocal images of a membrane tube pulled from HEK293T-GPMV containing TSPAN-GFP (green) and dyed with DiIC12 (red) aspirated at zero suction pressure (t=17 s). At t=20 s, the suction pressure was increased instantly to 0.25 mbar (corresponds to membrane tension of $\sigma = 4.1 \times 10^{-5}\ N/m$), which induced swellings formation on the tube. On the right, the force acting on the tube during the experiment. Apperence of swellings upon tension jump was observed 25 times in 20 membrane tubes pulled from 10 vesicles (HEK239T-TSPAN4-GPMVs) in 7 independent experiments.

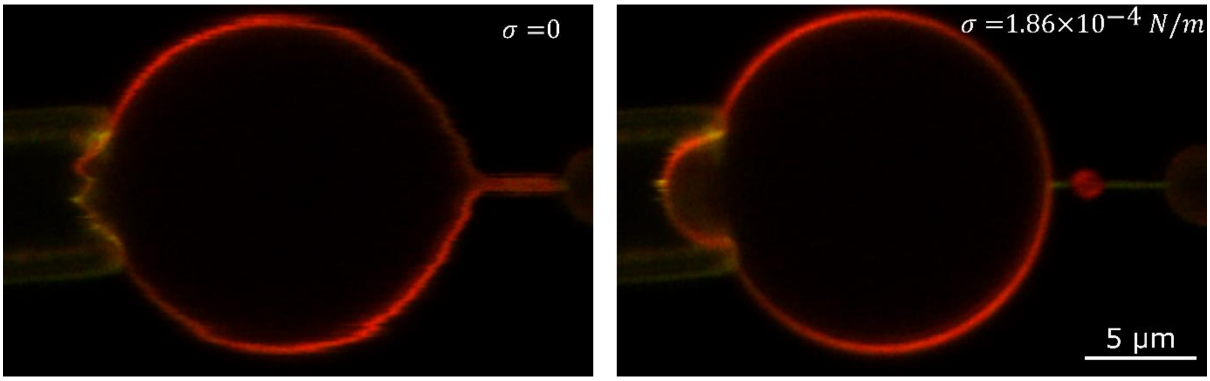

Figure S8. Swelling formation in NRK-GPMVs induced by tension increase. Confocal microscopy images of a membrane tube pulled from NRK-GPMV containing TSPAN-GFP (green) and dyed with DiIC12 (red). In the left image the zero suction pressure was zero (corresponds to zero tension applied, $\sigma = 0$). Next, the suction pressure was increased instantly to 1 mbar (corresponds to membrane tension of $\sigma = 1.86 \times 10^{-4}\ N/m$), which induced swellings formation on the tube. Apperence of swellings upon tension jump was observed 25 times in 19 membrane tubes pulled from 9 vesicles (NRK-TSPAN4-GPMVs) in 4 independent experiments.

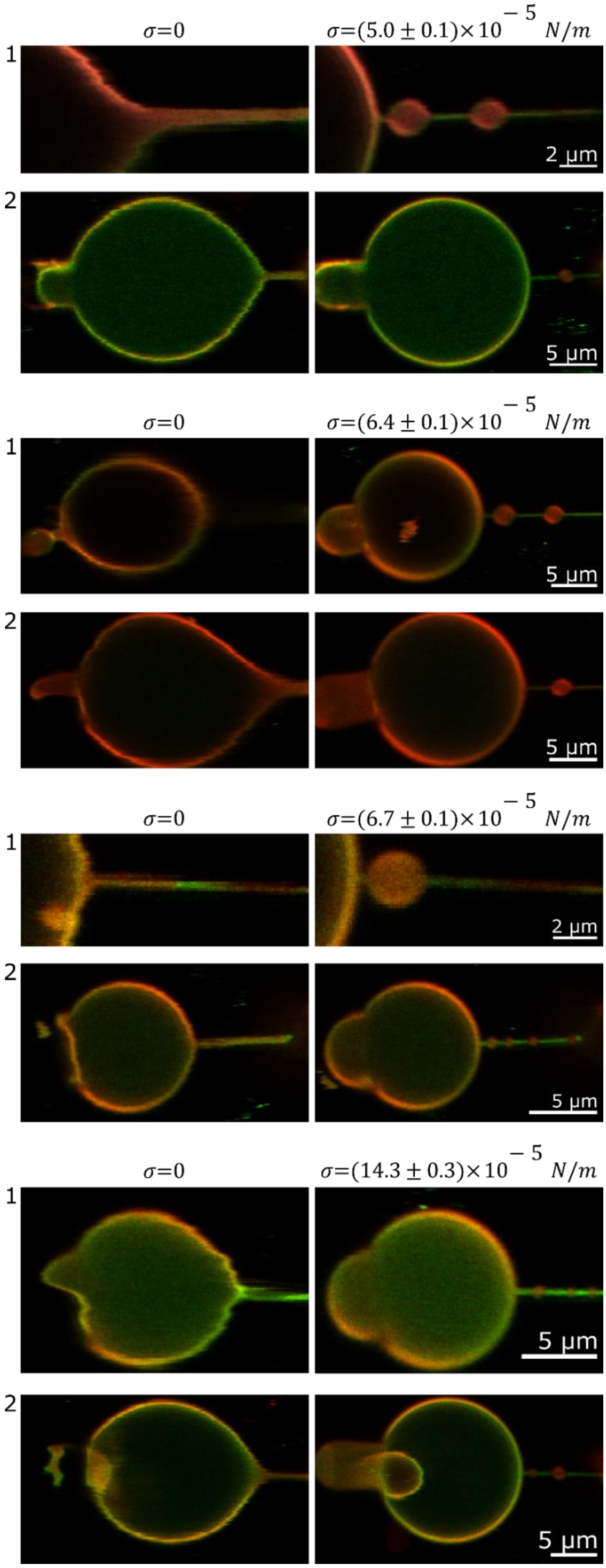

Figure S9. Initial swelling formation. Confocal microscopy images of a membrane tube pulled from HEK293T-GPMVs containing TSPAN-GFP (green) and dyed with DiIC12 (red). The figure includes 4 pairs of HEK293T-TSPAN4-GPMVs in which each pair was subjected to the same

tension jump (n=7 independent experiments). In the left images the suction pressure was zero (corresponds to zero tension applied, $\sigma = 0$). Next, the tension was increased instantly to the indicated value. It can be seen that same tension jumps generate different number of swellings in different positions along the tether.

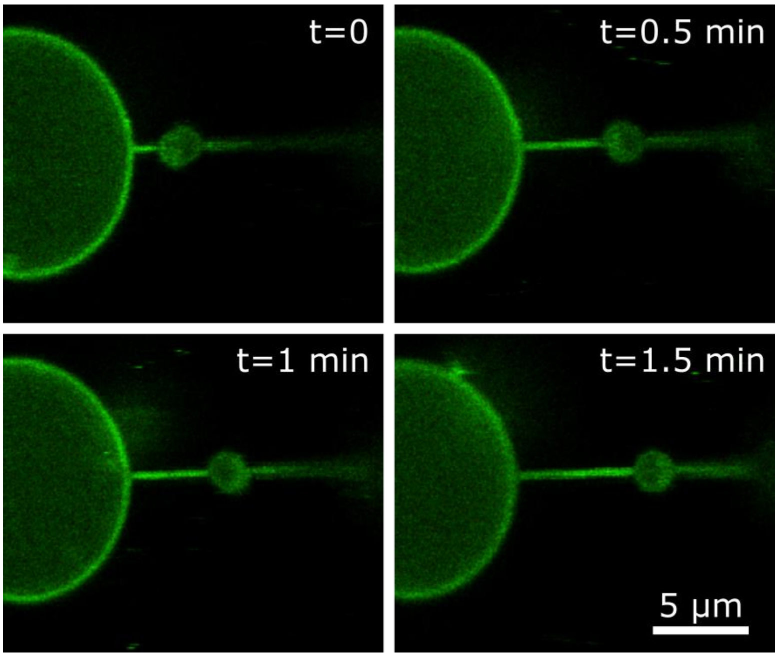

Figure S10. Swelling movement along membrane tube. Time-lapse confocal microscopy images of tube pulled from HEK293T-GPMV containing TSPAN4-GFP. After tension induced swelling formation, the swelling was able to move freely along the membrane tube. N=9 swellings on 9 membrane tubes pulled from 5 vesicles (HEK293T-TSPAN4-GPMVs) in 5 independent experiments.

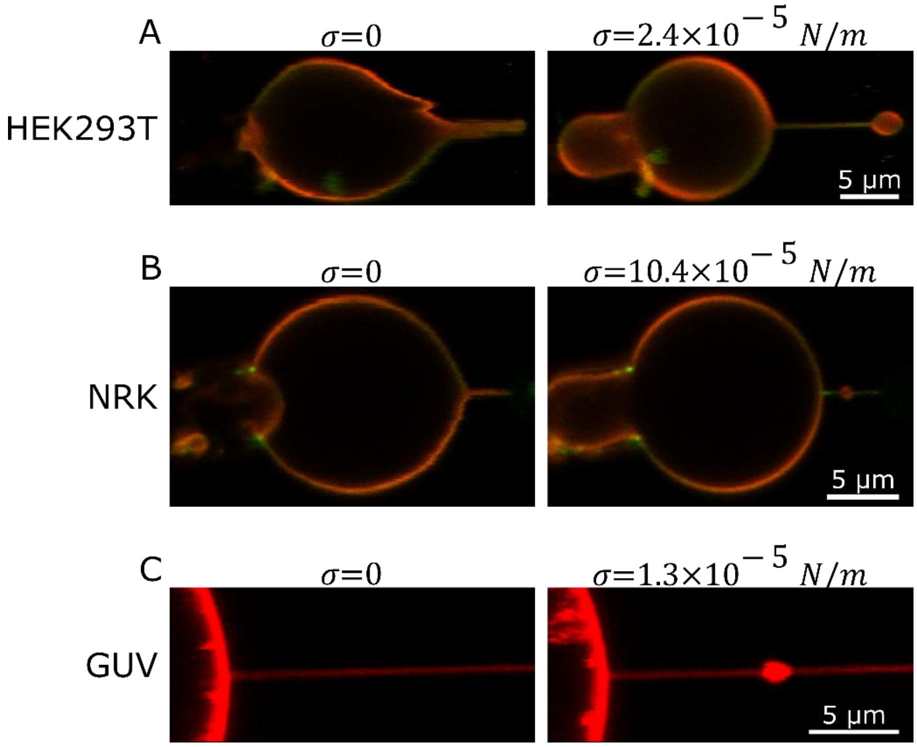

Figure S11. Tension threshold for swelling formation in membrane tubes in different membrane vesicles. (A) Confocal microscopy image of a membrane tube pulled from HEK293T-GPMVs containing TSPAN4-GFP (green) and DiIC12 (red).The lowest tension jump that formed a swelling was Δσ=0.024 mN/mN=25 swellings from 20 membrane tubes pulled from 10 vesicles (HEK293T-TSPAN4-GPMVs) in 7 independent experiments. (B) Confocal microscopy image of a membrane tube pulled from NRK-GPMVs containing TSPAN4-GFP (green) and DiIC12 (red). The lowest tension jump that formed a swelling was Δσ=0.104 mN/m N=25 swellings from 19 membrane tubes pulled from 9 vesicles (NRK-TSPAN4-GPMVs) in 4 independent experiments. (C) Confocal microscopy image of a membrane tube pulled from giant unilamellar vesicle (GUV) containing DOPC:DOPS:Rhodamine-PE, 90:9.9:0.1%. The lowest tension jump that formed a swelling wasΔσ=0.013 mN/m N=18 swellings on 13 membrane tubes pulled from 6 GUVs in 3 independent experiments.

It should be noted that GPMVs or GUVs from the same preparation had different tension threshold value for swelling formation. This might result from differences in membrane composition and bending rigidity, which can differ between GPMVs from the same cell culture[2]. The tension jumps appeared in the figure are the lowest jump that generate swelling in each type of vesicle.

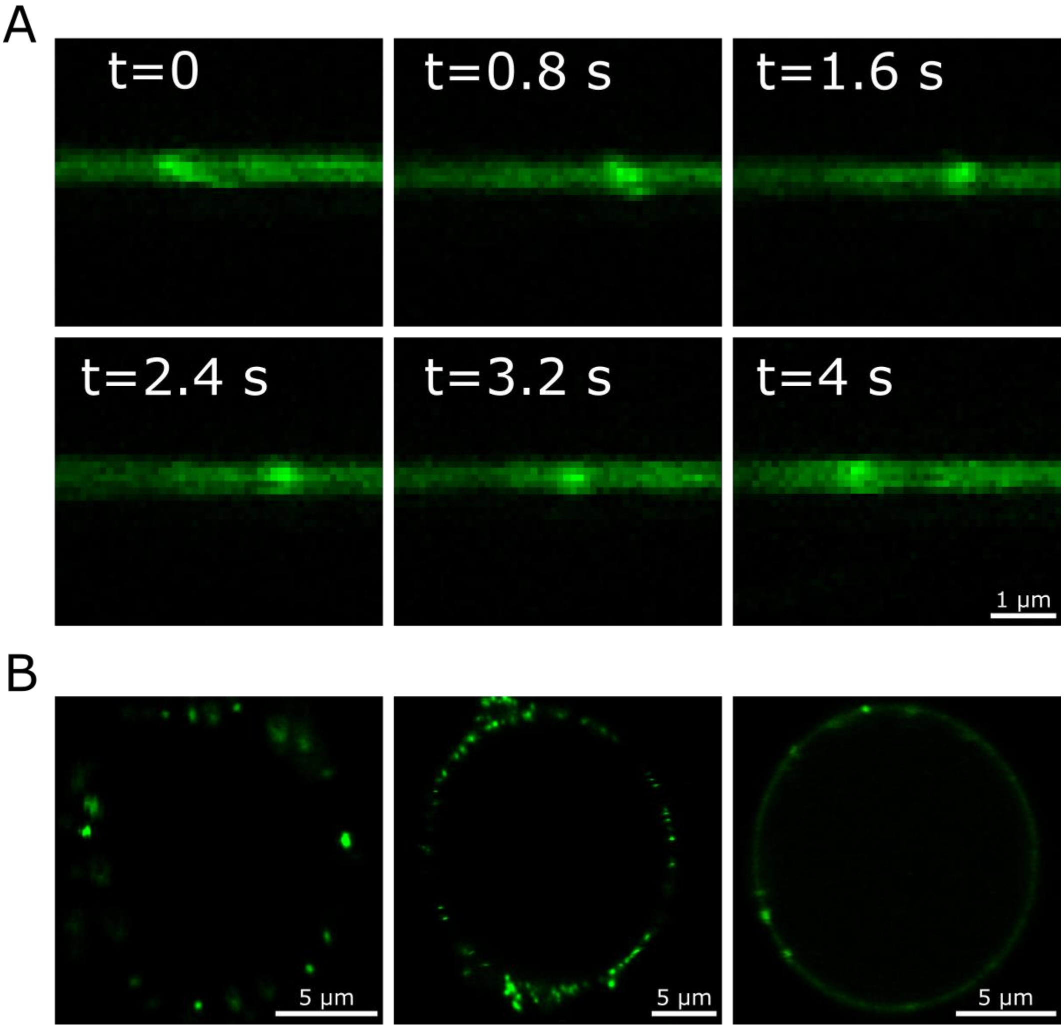

Figure S12. Formation of TSPAN4 domains induced by membrane curvature and shear forces. (A) Time-lapse confocal microscopy images of membrane tube enriched by TSPAN4-GFP at different time points as indicated. The first image (t=0) was taken 21 min after pulling the tube from HEK293T-GPMV. GFP puncta, which relates to TSPAN4 domains can be seen on the membrane tube. (B) Confocal microscopy images of HEK293T-GPMVs, containing TSPAN-GFP, after flowing through microfluidic channel. The images show TSPAN4 domains on the GPNVs, which can induce significant membrane remodeling. Most of the vesicles ruptured during the flow in the microfluidic channel (N= 2 independent experiments).

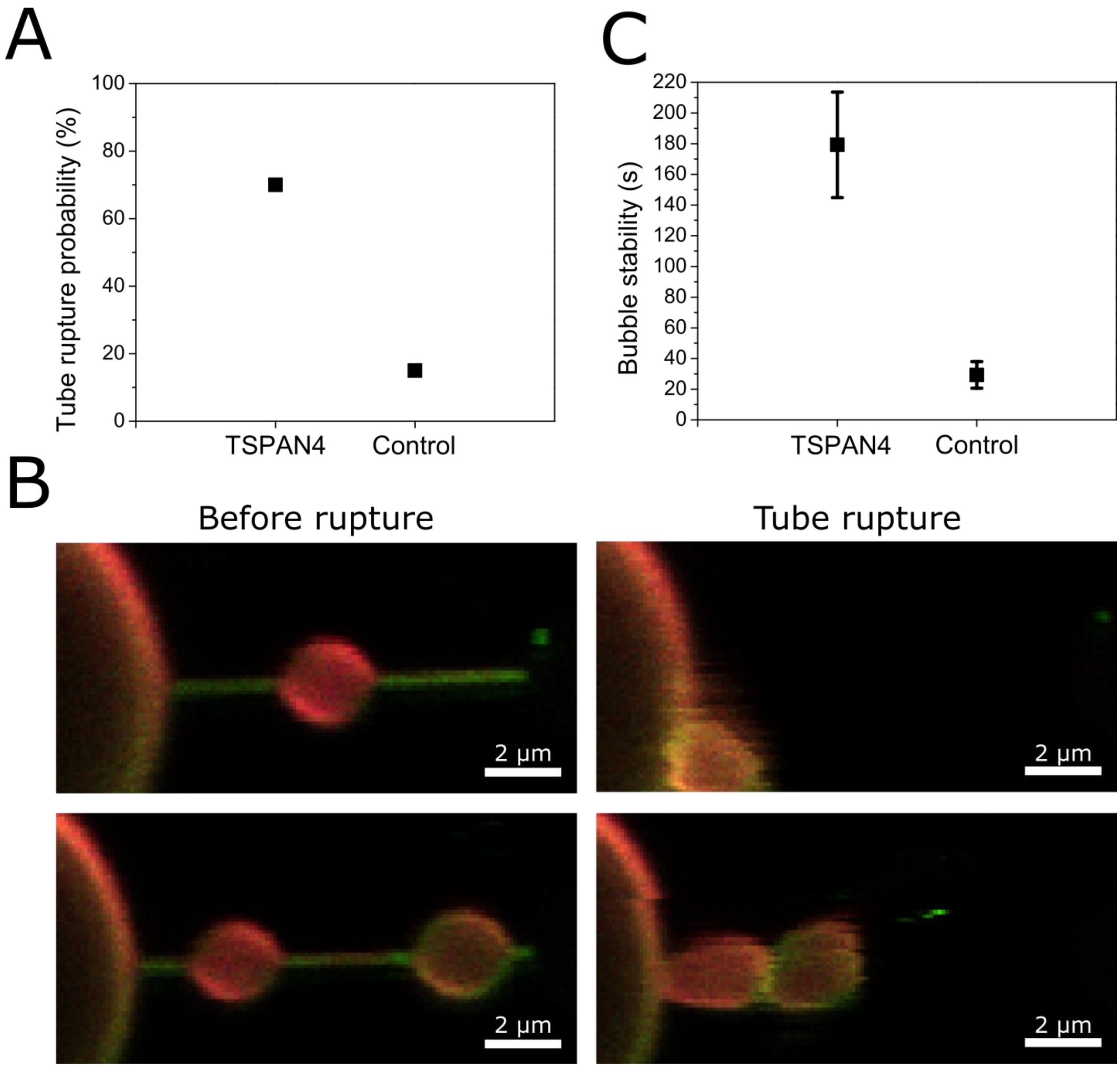

Figure S13. Swelling stability and tube rupture in HEK293T-GPMVs. (A) Percentage of tube rupture events with or without TSPAN4 (TSPAN4 n=20 membrane tubes pulled from 10 vesicles (HEK293T-TSPAN4-GPMVs) in 7 independent experiments, control n=20 membrane tubes pulled from 8 vesicles (HEK293T-GPMVs) in 3 independent experiments). (B) Confocal microscopy images of swellings on a membrane tube pulled from GPMV containing TSPAN4-GFP (green) and DiI-C12 (red). On the left, the last frame before tube rupture, and on the right, the frame right after the tube rupture. The images show that the swellings were intact after the rupture. (C) Swelling stability on membrane tubes with or without TSPAN4. In the presence of TSPAN4 in most of the experiments the swellings were stable, and experiments were stopped due to tube rupture. Hence, the stability of the swelling in the presence of TSPAN4 is probably higher than indicated. (TSPAN4 n=25 swellings on 20 membrane tubes pulled from 10 vesicles (HEK293T-TSPAN4-GPMVs) in 7 independent experiments), Control n=24 swellings on 20 membrane tubes pulled from 8 vesicles ((HEK293T-GPMVs) in 3 independent experiments). Error bars are SEM. Source data are provided as a Source Data file.

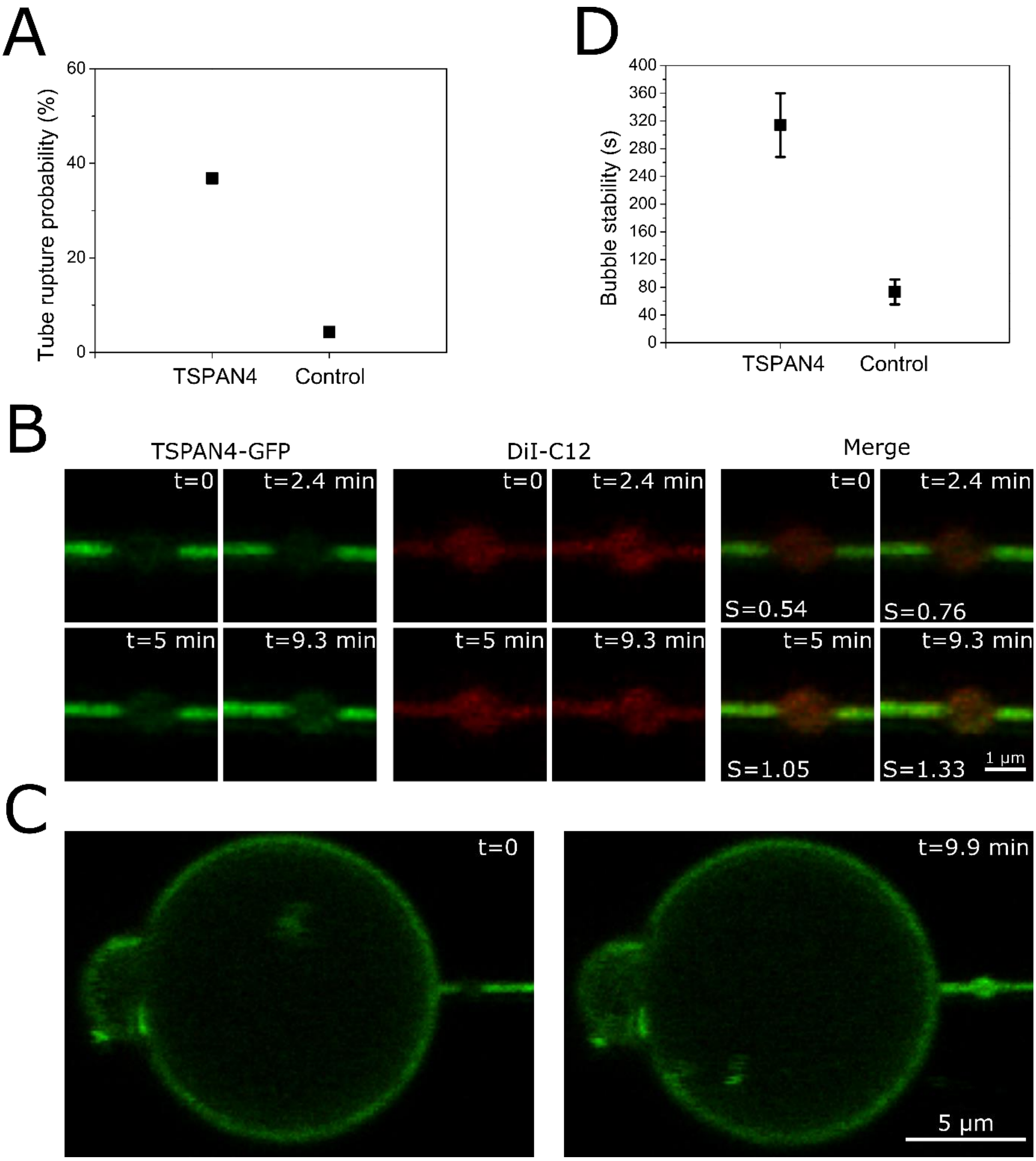

Figure S14. Swelling stability and TSPAN4 enrichment and tube rupture in NRK-GPMVs. (A) Percentage of tube rupture events with or without TSPAN4 (TSPAN4 n=19 membrane tubes pulled from 9 vesicles (NRK-TSPAN4-GPMVs) in 4 independent experiments, control n=47 membrane tubes pulled from 19 vesicles (NRK-GPMVs) in 5 independent experiments). (B) Time-lapse confocal microscopy images of swellings on a membrane tube pulled from GPMV containing TSPAN4-GFP (green) and DiI-C12 (red). The time (t) and soring factor (S) are indicated. (C) Confocal microscopy images of swellings on a membrane tube pulled from GPMV containing TSPAN4-GFP (green) after the initial swelling (t=o) and following TSAPN4 swelling recruitment (t=9.9 min). (D) Swellings stability on membrane tubes with or without TSPAN4. In the presence of TSPAN4 in most of the experiments the swellings were stable, and

experiments were stopped after 10 min. Hence, the stability of the swelling in the presence of TSPAN4 is probably higher than indicated. (TSPAN4 n=25 swellings on 19 membrane tubes pulled from 9 vesicles (NRK-TSPAN4-GPMVs) in 4 independent experiments, Control n=58 swellings on 47 membrane tubes pulled from 19 vesicles (NRK-GPMVs) in 5 independent experiments. Error bars are SEM. Source data are provided as a Source Data file.

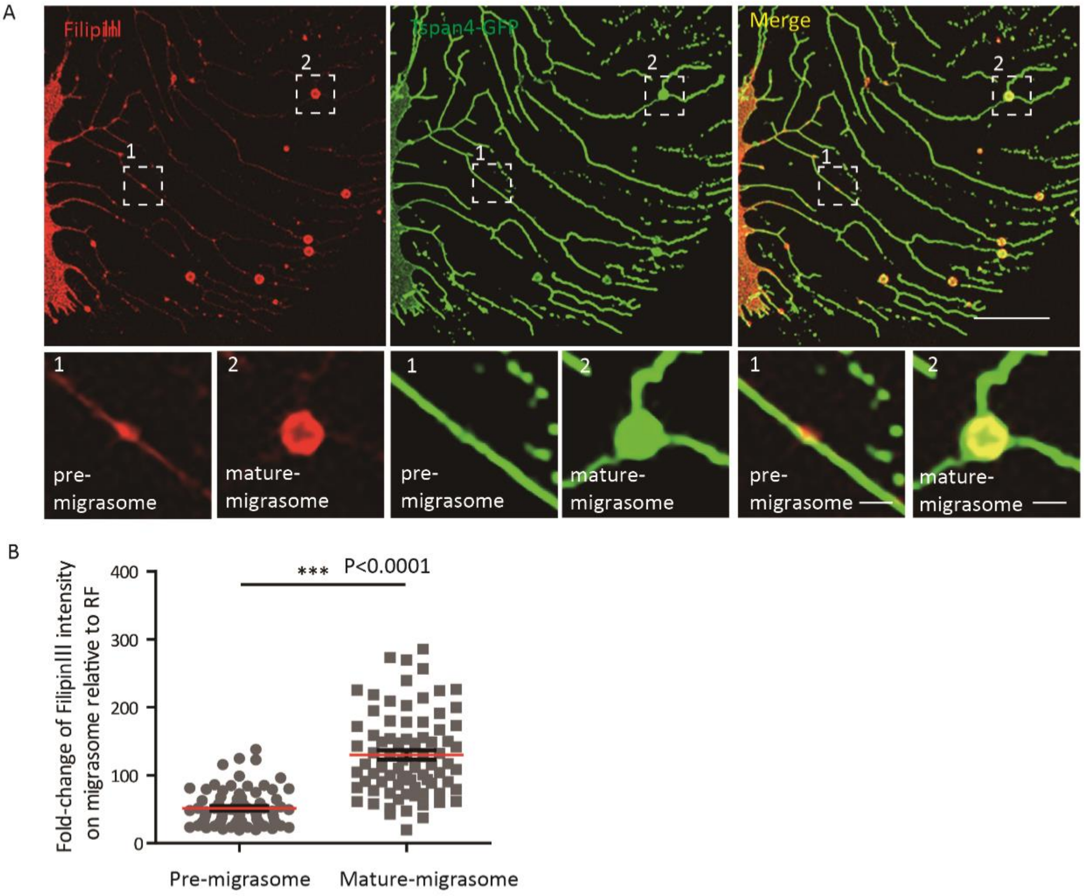

Figure S15. Cholesterol enrichment in pre and mature migrasomes. (A) Z-stack images of TSPAN4-GFP-expressing cells that were stained using filipin III. Scale bar, 10 µm; zoom in, 1µm. (B) Fold change of filipin III intensity on migrasome relative to retraction fiber. Based on series of images as in A, the following parameters were determined: filipin III intensity on migrasome, IpM; filipin III intensity on RF, IpR. Within a pair, the values of IpM/IpR on pre- and mature- migrasomes are displayed. Data shown represents the mean ± SEM. n =76 pairs of pre-migrasomes, n =79 pairs of mature migrasomes, from 3 independent experiments. Two-tailed unpaired t-tests were used for statistical analysis. P value= 5.5×10^{-18}. Source data are provided as a Source Data file.

Supplementary References

1. Raviv Dharan, Shahar Goren, Sudheer Kumar Cheppali, P. S. & Brand Guy, Li Yu, Michael M. Kozlov, and R. S. Transmembrane proteins tetraspanin 4 and CD9 sense membrane curvature. 1–15 (2022).

2. Steinkühler, J., Sezgin, E., Urbančič, I., Eggeling, C. & Dimova, R. Mechanical properties of plasma membrane vesicles correlate with lipid order, viscosity and cell density. *Commun. Biol.* **2**, 1–8 (2019).
