# Supplementary material for: Tetraspanin 4 stabilizes membrane swellings and facilitates their maturation into migrasomes
Source: Nat Commun. 2023 Feb 23;14:1037. doi: 10.1038/s41467-023-36596-9 (PMC9950420; doi:10.1038/s41467-023-36596-9)
Supplement: Supplementary file 2 — Description of Additional Supplementary files [file 41467_2023_36596_MOESM2_ESM.pdf]

Description of Additional Supplementary Files

File name: Supplementary Movie 1

Description: Movie of migrasomes formation of a NRK Tspan4-GFP cell stained by FM 4-64 conducted by confocal microscope at 1frame/4min speed. Green, Tspan4-GFP; red, FM 4-64; yellow, merge. Video display rate, 5 fps. Scale bar, 10 µm.

File name: Supplementary Movie 2

Description: Movie of migrasomes formation of a WT NRK cell stained by FM 4-64 conducted by confocal microscope at 1 frame/5min speed. red, FM 4-64. Video display rate, 10 fps. Scale bar, 5 µm.

File name: Supplementary Movie 3

Description: Movie of migrasomes formation of a NRK TSPAN4-GFP cell stained by FM 4-64 conducted by confocal microscope at 1 frame/5min speed. red, FM 4-64. Video display rate, 10 fps. Scale bar, 5 µm.

File name: Supplementary Movie 4

Description: Movie of migrasomes formation of a WT MGC803 cell stained by FM 4-64 conducted by confocal microscope at 1 frame/5min speed. red, FM 4-64. Video display rate, 10 fps. Scale bar, 5 µm.

File name: Supplementary Movie 5

Description: Movie of migrasomes formation of a MGC803 TSPAN4-KO cell stained by FM 4-64 conducted by confocal microscope at 1 frame/5min speed. red, FM 4-64. Video display rate, 10 fps. Scale bar, 5 µm.

File name: Supplementary Movie 6

Description: Bright field microscopy movie of swelling formation on membrane tube pulled from giant plasma membrane vesicle containing TSPAN4-GFP by increasing the membrane tension through increasing the suction pressure in the micropipette. Scale bar, 5 µm.
